# STAT3 Deficiency Alters the Macrophage Activation Pattern and Enhances Matrix Metalloproteinase 9 Expression during Staphylococcal Pneumonia

**DOI:** 10.4049/jimmunol.2300151

**Published:** 2023-11-20

**Authors:** Susan Farmand, Vicky Sender, Jens Karlsson, Padryk Merkl, Staffan Normark, Birgitta Henriques-Normark

**Affiliations:** * Department of Microbiology, Tumor and Cell Biology, Karolinska Institutet, Stockholm, Sweden; † Division of Pediatric Stem Cell Transplantation and Immunology, Clinic of Pediatric Hematology and Oncology, University Medical Center Hamburg-Eppendorf, Hamburg, Germany; ‡ Clinical Microbiology, Karolinska University Hospital Solna, Stockholm, Sweden

## Abstract

*Staphylococcus aureus* is a significant cause of morbidity and mortality in pulmonary infections. Patients with autosomal-dominant hyper-IgE syndrome due to STAT3 deficiency are particularly susceptible to acquiring staphylococcal pneumonia associated with lung tissue destruction. Because macrophages are involved in both pathogen defense and inflammation, we investigated the impact of murine myeloid STAT3 deficiency on the macrophage phenotype in vitro and on pathogen clearance and inflammation during murine staphylococcal pneumonia. Murine bone marrow–derived macrophages (BMDM) from STAT3 LysMCre^+^ knockout or Cre^−^ wild-type littermate controls were challenged with *S. aureus*, LPS, IL-4, or vehicle control in vitro. Pro- and anti-inflammatory responses as well as polarization and activation markers were analyzed. Mice were infected intratracheally with S. *aureus*, bronchoalveolar lavage and lungs were harvested, and immunohistofluorescence was performed on lung sections. *S. aureus* infection of STAT3-deficient BMDM led to an increased proinflammatory cytokine release and to enhanced upregulation of costimulatory MHC class II and CD86. Murine myeloid STAT3 deficiency did not affect pathogen clearance in vitro or in vivo. Matrix metalloproteinase 9 was upregulated in *Staphylococcus*-treated STAT3-deficient BMDM and in lung tissues of STAT3 knockout mice infected with *S. aureus*. Moreover, the expression of miR-155 was increased. The enhanced inflammatory responses and upregulation of matrix metalloproteinase 9 and miR-155 expression in murine STAT3-deficient as compared with wild-type macrophages during *S. aureus* infections may contribute to tissue damage as observed in STAT3-deficient patients during staphylococcal pneumonia.

## Key Points

- *S. aureus*–induced macrophage activation is enhanced in STAT3 deficiency.
- *S. aureus* augments MMP9, CD86, MHCII, and miR-155 expression in STAT3-deficient BMDM.
- Lack of myeloid STAT3 does not alter *S. aureus* killing but may affect tissue damage.

## Visual Abstract

**Figure:**
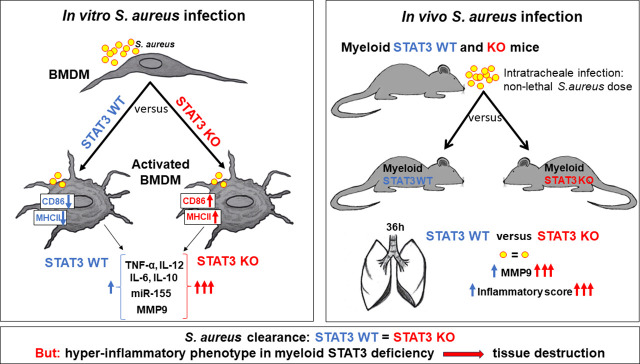

## Introduction

S*taphylococcus aureus* is a major pathogen associated with both healthy colonization and life-threatening invasive diseases such as pneumonia and septicemia (1). Pneumonia caused by *S. aureus* is rare in the immunocompetent host; yet, it poses a severe complication in patients with underlying conditions, especially in the nosocomial setting (2). During the last two decades, however, a rise has been observed of community-acquired staphylococcal pneumonia in otherwise healthy individuals due to the emergence of hypervirulent and often multiresistant strains (3, 4). Despite intensive efforts, there are still no commercial vaccines available. Efficient vaccine development is particularly hampered because *S. aureus* has a considerable potential for immune evasion, and essential defense mechanisms against *S. aureus* are predominantly dependent on innate immune function, which is more difficult to target with vaccines (5). Thus, a profound understanding of host–pathogen interactions during staphylococcal infection is of great importance.

Neutrophil dysfunction is a risk factor for staphylococcal infections, but less is known about the role played by macrophages during *S. aureus* infections (2). Macrophages display a high functional plasticity and may polarize into different phenotypes and even reverse their phenotype (6). The proinflammatory M1 phenotype and the repairing/immune modulatory M2 phenotype are regarded as the best characterized extremes of a wide spectrum of macrophage polarization (7, 8). A typical M1 inducer is LPS, which leads to release of IL-12, upregulation of inducible NO synthase (iNOS), and in dependence on the presence of other signals (e.g., IFN-γ) also to increased Ag presentation via MHC class II (MHCII) upregulation (9, 10). In contrast, M2-type macrophages produce high levels of anti-inflammatory IL-10 and low levels of proinflammatory cytokines. They are generally observed in healing-type circumstances in the wake of an infection but may also occur due to other causes. The M2-type responses can be further augmented and shaped by cytokines such as IL-4, IL-10, or IL-13 (11). Murine M1 macrophages produce high levels of NO and reactive oxygen species (ROS), whereas M2 macrophages produce ornithine and polyamines via the arginase pathway and show upregulation of chitinase-3-like protein 3 (YM1; a member of the chitinase family) and found in inflammatory zone 1 (FIZZ1; RETNLA) (11, 12). While proinflammatory M1 macrophages are linked to Th1-type immune responses and pathogen killing, M2 macrophages are associated with Th2-type responses, immune tolerance, tissue remodeling, and tumor progression (9, 13). Infection-induced inflammation is pivotal in the initial combat against infectious pathogens. Yet, an overwhelming proinflammatory phenotype in the later phase of infection may ultimately deteriorate the outcome and enhance tissue damage, as observed in the context of M1 persistence (14).

Reduced function of STAT3 in patients with autosomal-dominant hyper-IgE syndrome (STAT3-deficient HIES) increases the susceptibility to *S. aureus* pneumonia and is also frequently associated with postinfectious lung tissue damage (15, 16). STAT3 is an ubiquitously expressed transcription factor that is among others involved in signal transduction of a wide range of different cytokines. Notably, predominantly proinflammatory IL-6 and anti-inflammatory IL-10 both have their key downstream signaling via STAT3 (17). STAT3 deficiency leads to an enhanced signature of both pro- and anti-inflammatory cytokines, and downstream signaling of these cytokines may involve both STAT3-dependent and STAT3-independent pathways (18). Complete STAT3 deficiency is embryonically lethal, and patients with STAT3-deficient HIES have residual signaling (19). The degree of maintained STAT3 signaling and somatic mosaicism likely accounts for phenotypic differences between individual patients (20). Deciphering the role of STAT3 on a specific disease entity is hampered by the pleiotropic effect of this transcription factor (17).

The matrix metalloproteinase (MMP) family, such as MMP9, is involved in inflammation and tissue restructuring and plays important roles in the pathogenesis of several lung diseases (21). The functional levels of MMPs depend on their level of transcription/translation, on the proteolytic cleavage of their latent proforms (e.g., pro-MMP9), as well as on levels of their endogenous tissue inhibitors (tissue inhibitor of metalloproteinase [TIMP]) (22). MicroRNAs (miR), such as miR-155, are small noncoding RNAs that regulate gene expression post-transcriptionally and play important roles in regulating inflammatory, tissue damaging, and repair processes (23).

In this study, we investigated the pro- and anti-inflammatory effects of staphylococcal infection of bone marrow–derived macrophages (BMDM) from myeloid STAT3 knockout (KO) mice compared with wild-type (WT) mice. We found that stimulation with *S. aureus* induced a unique macrophage phenotype displaying both M1- and M2-like features. STAT3 deficiency skewed the macrophage phenotype to a proinflammatory M1-like phenotype, but pathogen clearance was not affected. *S. aureus* stimulation of STAT3-deficient BMDM led to defective IL-10 and IL-6 signaling and an upregulation of miR-155. Furthermore, MMP9 was upregulated, both in *S. aureus*-infected STAT3-deficient BMDM and in lung tissues from myeloid STAT3-deficient mice infected with *S. aureus*. The observed inflammatory signatures likely affect the degree of tissue destruction.

## Materials and Methods

### Mouse model

STAT3LysMCre^+^ (STAT3 KO) and STAT3LysMCre**^−^** (STAT3 WT) mice were bred under specific pathogen-free conditions. The STAT3^flox^ mice were originally purchased from The Jackson Laboratory. Myeloid-specific loss of STAT3 was achieved by breeding with transgenic *LysMCre* mice (24). All experiments were performed in accordance with the local ethical committee (Stockholms Norra djurförsöksetiska nämnd). Female and male mice between 6 and 12 wk of age were used for experiments. Samples obtained from STAT3 KO mice were concomitantly processed with at least one STAT3 WT littermate control sample.

### In vitro infection model using BMDM

BMDM were generated by harvesting bone marrow cells from STAT3 WT and KO mice followed by 6-d incubation in DMEM (with phenol red and l-glutamine; Life Technologies), 30% M-CSF containing L929-derrived medium, 10% FCS (Life Technologies), 1% penicillin-streptomycin (Thermo Fisher Scientific). Then, BMDM were harvested and reseeded for stimulation experiments. Stimulations of BMDM were performed on day 7 in RPMI 1640 without phenol red (Sigma-Aldrich), 10% FCS (Life Technologies), 2 mM l-glutamine (Life Technologies). This medium was also used as a vehicle control (vcl). Nonstimulated cells showed the classical BMDM appearance on light microscopy (Supplemental Fig. 1A), and nearly all cells were F4/80^+^ CD11b^+^ (Supplemental Fig. 1B). The following stimulations were used: 100 ng/ml LPS (Sigma-Aldrich) as an M1 phenotype inducer, 20 ng/ml IL-4 (PeproTech) as an M2 phenotype inducer, and live *S. aureus* 8325-4 (multiplicity of infection [MOI] 10). After 2 h, penicillin-streptomycin (Thermo Fisher Scientific) was added to all wells to stop bacterial growth. At 24 or 48 h after stimulation, supernatants were harvested and samples were processed for various analyses.

### Flow cytometry

After the harvest of cell-free supernatants at 24 h, BMDM were washed and blocked with Fc block (Fc block CD16/CD32, AH Diagnostics). Then, fixable viability dye (FVD 780, AH Diagnostics) and surface staining (MHCII Alexa Fluor 700, F4/80 PE, CD80 eFluor 450, CD86 PE-Cy7, all AH Diagnostics; and CD11b PE-CF594, BD Biosciences) were performed. In addition, internal expression of iNOS Alexa Fluor 488 (AH Diagnostics) and arginase 1 (Arg1) allophycocyanin (R&D Systems) was determined following fixation with 2% paraformaldehyde and permeabilization with Perm buffer (AH Diagnostics). Flow cytometric analysis was performed on a Gallios flow cytometer, and Kaluza software (Beckman Coulter) was used for analysis (Supplemental Fig. 1B–1E).

### ELISA

TNF-α, IL-6, IL-10, and IL-12p70 (BD Biosciences), as well as pro-MMP9 and TIMP1 (Thermo Fisher), were measured in the cell-free supernatant by ELISA according to the manufacturer’s instructions.

### NO assay

Supernatants of stimulated BMDM were analyzed for NO with a commercial NO assay (Spectroquant) using the Griess reaction. Absorbance was measured at 525 nm using a SpectraMax Plus 384 microplate reader (Molecular Devices).

### RNA isolation, cDNA, and qPCR of BMDM

In selected experiments, following the harvest of supernatants, cells were washed with PBS, and TRIzol (Life Technologies) was added for cell lysis and subsequent RNA retrieval. RNA was purified by using chloroform and separation in Phase Lock Gel heavy tubes (5Prime), followed by precipitation with isopropanol and ethanol. The initial purity and amount were estimated by NanoDrop (Thermo Scientific). DNA digestion was performed using TURBO DNase buffer (Ambion). For generation of cDNA, the high-capacity cDNA reverse transcription kit (Applied Biosystems) was employed according to the manufacturer’s instructions. PCR and cDNA reactions were performed on the S1000 Thermal Cycler (Bio-Rad Laboratories). Real-time quantitative PCR by using SYBR Green detection (Bio-Rad Laboratories) was performed on a CFX Connect Real-Time PCR Detection System (Bio-Rad Laboratories). Primer sequences of target genes and references are listed in Supplemental Table I. Analysis was performed by applying the ΔΔCq method. Expression at 24 h was normalized to β-actin and β_2_-microglobulin, and expression at 48 h was normalized to β-actin and hydroxymethylbilane synthase because these proteins proved stable expression at the respective time points (M-values <0.5).

### miR-155 quantification

Quantification of miR-155 was performed according to the manufacturer’s instructions using the TaqMan microRNA reverse transcription kit (Life Technologies) to create DNA from the microRNA (S1000 Thermal Cycler, Bio-Rad Laboratories) and then the TaqMan small RNA assay kit (Life Technologies) for the qPCR reaction (CFX Connect Real-Time PCR Detection System, Bio-Rad Laboratories). The amount of reverse-transcribed RNA was 10 ng. miR-155 expression was normalized to sno-202 expression.

### Staphylococcal uptake and killing assays in BMDM

For determination of early killing and uptake, BMDM were incubated with *S. aureus* (MOI 10) for 2 h at 37°C, 60 rpm (Minitron incubation shaker). For determination of internal bacteria, gentamicin was added to selected wells to kill external bacteria, and bacterial CFU were determined after cell lysis with saponin. Total bacteria and external bacteria were determined by serial dilution plating in other wells without gentamicin and with or without cell lysis, respectively. For late killing, external *S. aureus* was killed by gentamicin (50 µg/ml concentration) at 2 h followed by washing. After 24 h, cells were lysed with saponin and CFU were determined by serial dilution plating.

### In vivo staphylococcal pneumonia model

In order to assess the role of myeloid STAT3 deficiency in staphylococcal pneumonia, we set up an in vivo murine infection model. STAT3 KO and WT mice were anesthetized by i.p. injection of ketamine/xylazine and infected intratracheally with a sublethal dose of S. *aureus* 8325-4 (∼5 × 10^6^ CFU) or PBS (uninfected). Mice were followed for 12 or 36 h. Then, bronchoalveolar lavage (BAL) was performed by cannulating the trachea and flushing the lungs with 2 ml PBS + 0.2 mM EDTA. Afterward, lungs were harvested. In some experiments, the removed lungs were fixed in 10% formalin without prior performance of BAL and sent to the Swedish National Veterinary Institute (Statens Veterinärmediciniska Anstalt) for further processing for histological analysis.

### Analysis of murine BAL and lung samples

Lung cell suspensions were prepared by passing lung samples through a cell strainer (100 µm, Corning), followed by rinsing and resuspending in 1 ml PBS. After taking aliquots for CFU determination via serial dilution, the lung suspensions and BAL were centrifuged. Supernatants were filtered to eliminate remaining bacteria (0.22 µm) and stored at −80°C for later analysis. RBCs in the cell pellets were lysed with ammonium chloride followed by washing and resuspension with PBS. Total cell numbers were counted with a cell counter (Luna, Logos Biosystems). The cell suspensions were aliquoted and further processed for downstream applications. Diverse cell populations in lung and BAL samples were determined by multicolor flow cytometry.

### Flow cytometry of BAL and lung samples

Lung and BAL samples were incubated with live/dead staining dye FVD780 (eBioscience) followed by washing with PBS. Then, samples were fixed with 2% paraformaldehyde. After washing, the samples were blocked with Fc block (BD Biosciences) for 10 min on ice. Next, cell suspensions were stained for the surface Ags Ly6G FITC, Ly6c v450, NK1.1 allophycocyanin, CD11c PE-Cy7, CD11b PE-CF594, F4-80 PE, and MHCII Alexa Fluor 700. Respective isotype Abs (Iso ham PE-Cy7, IgG2b PE, IgG2a allophycocyanin, IgM v450) were used to set negative gates. Samples were run on a Gallios flow cytometer, and analysis was performed with Kaluza software (Supplemental Fig. 2).

### Histology of lung tissue samples

Slides for histology were prepared from paraffin-embedded tissue by the histology department of the Swedish National Veterinary Institute or by the core facility of Karolinska Institutet for immunohistology. Routine histological analysis and scoring were performed by H&E staining by a pathologist in a blinded fashion.

### MMP9 immunofluorescence in lung tissues

Slides from paraffin-embedded lung tissues were deparaffinized, and Sudan Black was used to reduce autofluorescence. Tris-EDTA buffer was used for Ag retrieval, and permeabilization was achieved by using 1% Triton X. Following blocking with 2% BSA in TBST (TBS + 0.1% Tween 20), tissues on slides were incubated with MMP9 Ab (Abcam, host rabbit) in 1% BSA in TBST overnight at 4°C. After washing, the tissues were incubated with anti-rabbit 488 Ab in 1% BSA in PBS for 1 h at room temperature, followed by washing and counterstaining with DAPI. Ultimately, coverslips were mounted with use of mounting medium. Images were taken with a DeltaVision microscope (20× objective, XY 2048 × 2048) using SoftWoRx software. Analysis was performed with ImageJ with the use of three standardized macros (Supplemental Macro Codes 1–3; https://doi.org/10.6084/m9.figshare.21930690.v1).

### Statistics

Statistical analysis was performed using GraphPad Prism 9. A mixed-effects model with Bonferroni analysis for multiple comparisons was used for statistical analysis if more than two variables were present. For data with two variables, the Mann–Whitney *U* test was used. Correlation was estimated using Pearson correlation.

## Results

### STAT3 deficiency enhances inflammatory responses and increases expression of costimulatory proteins in BMDM in response to *S. aureus* infection

**FIGURE 1.**
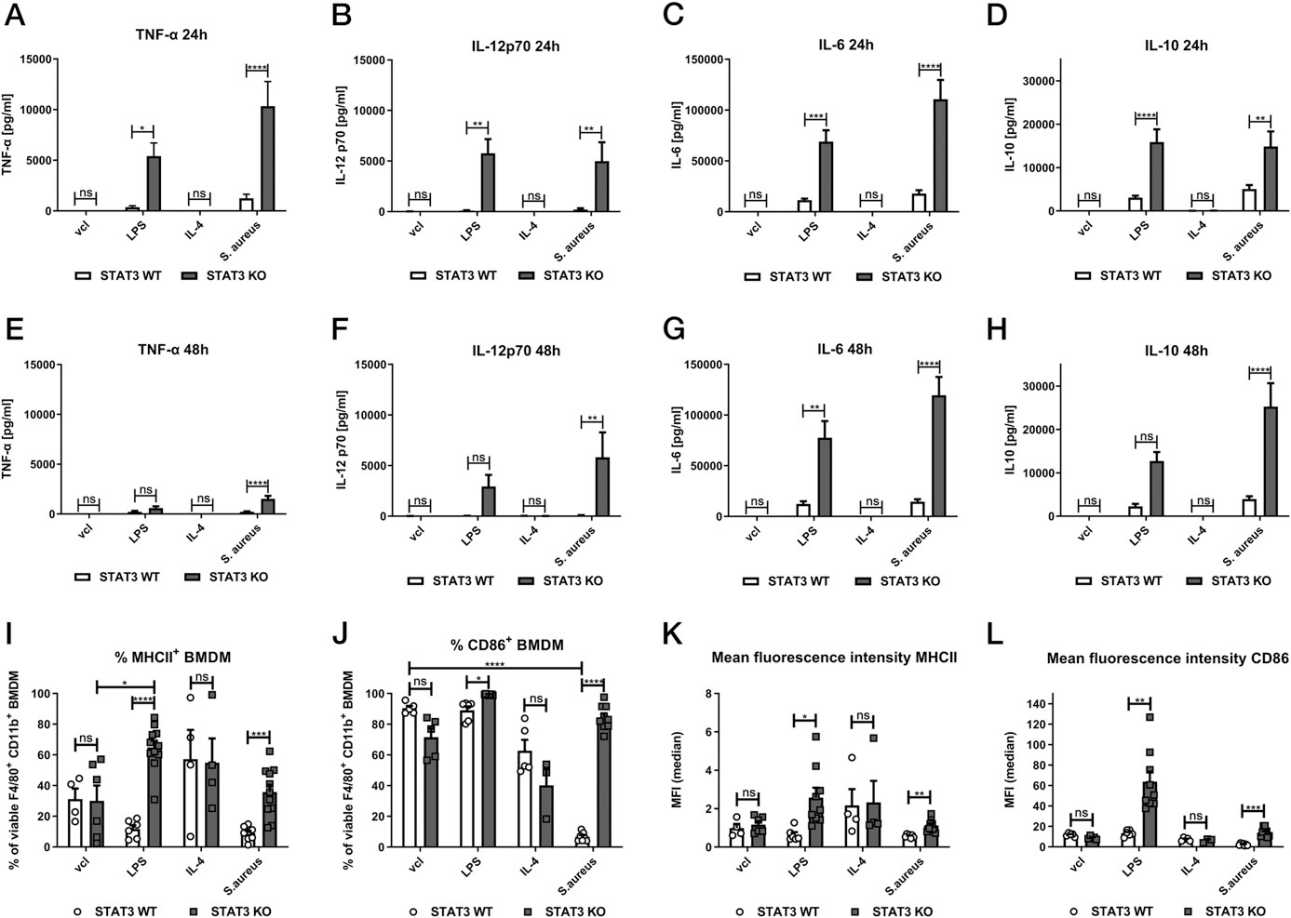
Enhanced inflammatory cytokine release and upregulation of costimulatory MHCII and CD86 in STAT3-deficient BMDM upon stimulation with *S. aureus*. BMDM from STAT3 WT and KO mice were stimulated with vcl, LPS (100 ng/ml), IL-4 (20 ng/ml), and *S. aureus* (MOI 10). At 2 h of stimulation, penicillin-streptomycin was added to kill viable *S. aureus*. After 24 or 48 h, supernatants were harvested and measured for different cytokines (TNF-α, IL-12p70, IL-6, IL-10) by ELISA. (**A**–**D**) Cytokine levels 24 h and (**E**–**H**) 48 h after stimulation. Pooled data (mean with SEM) from *n* ≥ 3 independent experiments are shown. (**I**–**L**) For flow cytometric analysis, live/dead staining as well as surface marker staining of BMDM was performed at 24 h. Gating was performed on single viable F4/80^+^ CD11b^+^ cells. Percentage of (I) MHCII^+^ and (J) CD86^+^ F4/80^+^ CD11b^+^ BMDM. (**K** and **L**) Mean fluorescence intensity (MFI) of (K) MHCII and (L) CD86 of F4/80**^+^** CD11b**^+^** BMDM. Pooled data (mean with SEM) from *n* ≥ 4 independent experiments are shown. Mixed-effects model with Bonferroni analysis for multiple comparisons was used for statistical analysis. *p* > 0.05 not significant; *p ≤ 0.05, **p ≤ 0.01, ***p ≤ 0.001, ****p ≤ 0.0001).

BMDM derived from STAT3-deficient and WT mice were stimulated with *S. aureus*, LPS, IL-4 or vehicle (unstimulated), and pro- and anti-inflammatory cytokines were measured. Release of proinflammatory TNF-α, IL-12p70, and IL-6 and anti-inflammatory IL-10 was strongly enhanced in STAT3-deficient BMDM challenged with *S. aureus* or LPS at 24 h (Fig. 1A–1D) and 48 h (Fig. 1E–1H) after challenge. Cytokine kinetics differed, with lower expression of TNF-α at 48 h compared with 24 h even for the STAT3-deficient BMDM upon both LPS and *S. aureus* stimulation. In contrast, *S. aureus*–stimulated production of IL-12 and IL-6 remained rather unchanged during the observation time, whereas IL-10 release was even higher at the 48-h time point in STAT3-deficient BMDM.

Moreover, MHCII expression was significantly increased in STAT3-deficient as compared with WT BMDM when exposed to *S. aureus* or LPS (Fig. 1I). CD86 was expressed on almost all F4/80**^+^** CD11b**^+^** WT and STAT3-deficient BMDM in the unstimulated state. However, in contrast to LPS stimulation, infection with *S. aureus* led to a significant reduction of CD86**^+^** cells to, on average, less than 15% in WT BMDM (Fig. 1J). This downregulation of CD86 was completely abrogated in STAT3-deficient BMDM. On average, over 80% of STAT3-deficient BMDM stained positive for CD86 after *S. aureus* stimulation. The mean fluorescence intensity of MHCII (Fig. 1K) and CD86 (Fig. 1L and Supplemental Fig. 1D, 1E) was enhanced in both LPS- and *S. aureus*–treated STAT3 KO samples. CD86 mRNA expression showed a trend toward higher values at 24 and 48 h in LPS-treated STAT3-deficient BMDM, whereas *Staphylococcus*-treated samples overall had low CD86 mRNA expression (Supplemental Fig. 1F, 1G).

**FIGURE 2.**
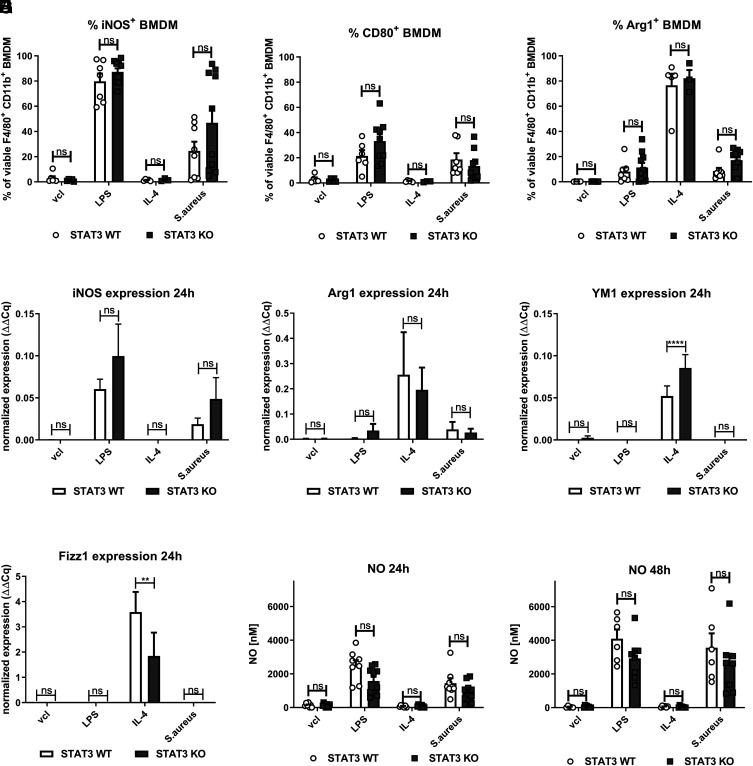
No changes in classical M1/M2 markers and NO induction in STAT3-deficient compared with WT BMDM upon *S. aureus* stimulation. STAT3 WT and KO BMDM were stimulated with vcl, LPS (100 ng/ml), IL-4 (20 ng/ml), or *S. aureus* (MOI 10) for a total of 24 or 48 h following addition of penicillin-streptomycin at 2 h of stimulation. Supernatants were harvested, and cells were further processed for downstream analysis. (**A**–**C**) For flow cytometric analysis of BMDM, live/dead staining, as well as surface marker staining (CD80), followed by permeabilization and intracellular staining (iNOS, Arg1), was performed at 24 h. Gating was performed on single viable F4/80^+^ CD11b^+^ BMDM. Percentage of (A) iNOS^+^, (B) CD80^+^, and (C) Arg1^+^ F4/80^+^ CD11b^+^ BMDM. (**D**–**G**) In selected experiments, cells were processed for RNA extraction by the TRIzol method. Following cDNA generation from RNA, qPCR of (D) iNOS, (E) Arg1, (F) YM1, and (G) Fizz1 was run. Pooled data (mean with SEM) from *n* ≥ 3 different experiments are shown. (**H** and **I**) NO measurement in supernatants from STAT3 WT and KO BMDM stimulated with vcl, LPS, IL-4, or *S. aureus* for (H) 24 and (I) 48 h. Pooled data (mean with SEM) from *n* ≥ 5 different experiments are shown. Mixed-effects model with Bonferroni analysis for multiple comparisons was used for statistical analysis. *p* > 0.05 not significant; **p ≤ 0.01, ****p ≤ 0.0001.

In addition, we studied expression of iNOS and CD80 and found that WT and STAT3-deficient BMDM showed similar increased levels upon LPS stimulation. IL-4 induced similar Arg1, but more YM1 and less Fizz1, expression in STAT3-deficient BMDM compared with the WT (Fig. 2). Stimulation with *S. aureus* also induced iNOS and CD80 expression and led to a minor Arg1 expression. Both LPS and *S. aureus* triggered release of NO (Fig. 2 H, 2I). However, no significant differences were observed between STAT3-deficient and WT BMDM for any of these markers upon LPS or *S. aureus* treatment. Furthermore, *S. aureus*–induced release of IL-1β did not vary depending on STAT3, and IFN-γ was not detected in *S. aureus*–treated STAT3 KO and WT BMDM at 24 h (Supplemental Fig. 3A, 3B).

### MMP9 is induced by *S. aureus* in STAT3-deficient BMDM

**FIGURE 3.**
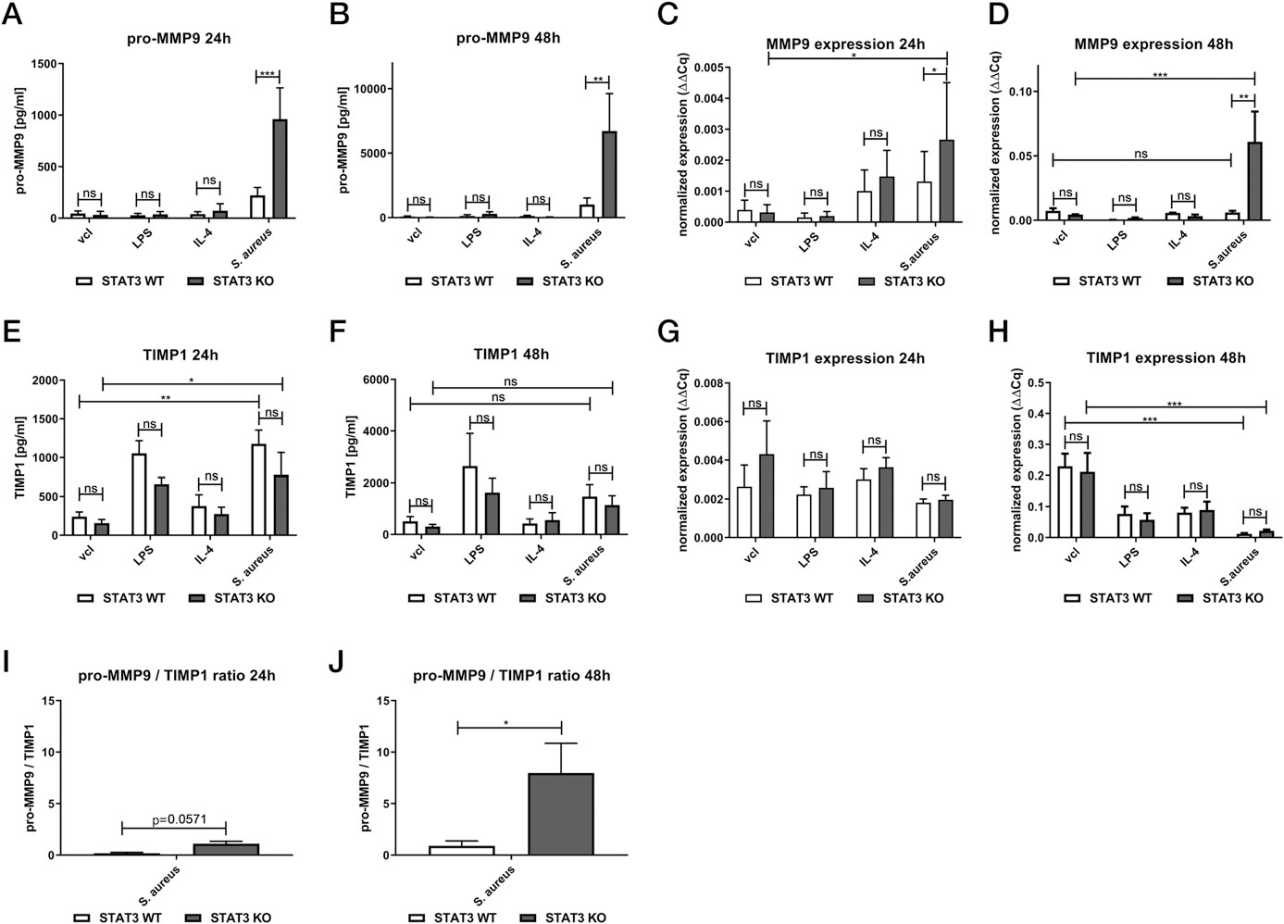
Enhanced induction of MMP9 in STAT3-deficient BMDM upon stimulation with *S. aureus*. STAT3 WT and KO BMDM were stimulated with vcl, LPS (100 ng/ml), IL-4 (20 ng/ml), or *S. aureus* (MOI 10). During the stimulation, penicillin-streptomycin was added at 2 h to all conditions to kill off viable *S. aureus*. At 24 or 48 h, supernatants were harvested for cytokine analysis by ELISA. In selected experiments, BMDM were subjected to RNA extraction by the TRIzol method. After generation of cDNA, qPCR analysis was performed. (**A** and **B**) Pro-MMP9 levels by ELISA at (A) 24 h and (B) 48 h after stimulation. (**C** and **D**) MMP9 RNA expression at (C) 24 h and (D) 48 h. (**E** and **F**) TIMP1 levels by ELISA at (E) 24 h and (F) 48 h after stimulation. (**G** and **H**) TIMP1 RNA expression at (G) 24 h and (H) 48 h. (**I** and **J**) Calculated ratio of pro-MMP9/TIMP1 protein levels by ELISA at (I) 24 h and (J) 48 h. Pooled data (mean with SEM) from *n* ≥ 3 different experiments are shown. (A–H) All data with more than two variables were analyzed by employing the mixed-effects model with Bonferroni analysis for multiple comparisons. (I and J) Pro-MMP9/TIMP1 protein ratio was analyzed with a nonparametric Mann–Whitney test. *p* > 0.05 not significant; *p ≤ 0.05, **p ≤ 0.01, ***p ≤ 0.001.

Because MMP9 has been reported to be elevated in the plasma of STAT3-deficient patients (25) and to play an important role in degradation of extracellular matrix and tissue remodeling, we then studied the expression of MMP9 in BMDM. Stimulation with *S. aureus* led to induction and release of pro-MMP9 into the supernatant at 24 and 48 h of WT BMDM (Fig. 3A, 3B), which was markedly enhanced in BMDM lacking STAT3. MMP9 RNA levels were also increased in STAT3-deficient BMDM stimulated with *S. aureus* at both 24 h (Fig. 3C) and 48 h (Fig. 3D). Challenge with LPS only induced pro-MMP9 release marginally in both WT and STAT3-deficient BMDM.

Next, we analyzed the induction of the inducer of TIMP1, which may counteract MMP9 activation (26). We observed significantly elevated levels of TIMP1 in *S. aureus*–stimulated samples, regardless of STAT3 at 24 h, but not at 48 h (Fig. 3E, 3F). At 48 h, TIMP1 was still measurable in the supernatant of all samples. Yet, following staphylococcal treatment, TIMP1 RNA at 48 h was downregulated compared with the control, vcl-treated sample at this time point, and no differences were seen depending on the presence of STAT3 (Fig. 3G, 3H).

Finally, the ratio between pro-MMP9/TIMP1 protein levels in the supernatant of *S. aureus*–stimulated samples was elevated in STAT3-deficient compared with WT BMDM (Fig. 3I, 3J), in particular at 48 h (Fig. 3J) after infection.

### Staphylococcal pneumonia is associated with elevated MHCII expression of alveolar macrophages in STAT3-deficient mice

**FIGURE 4.**
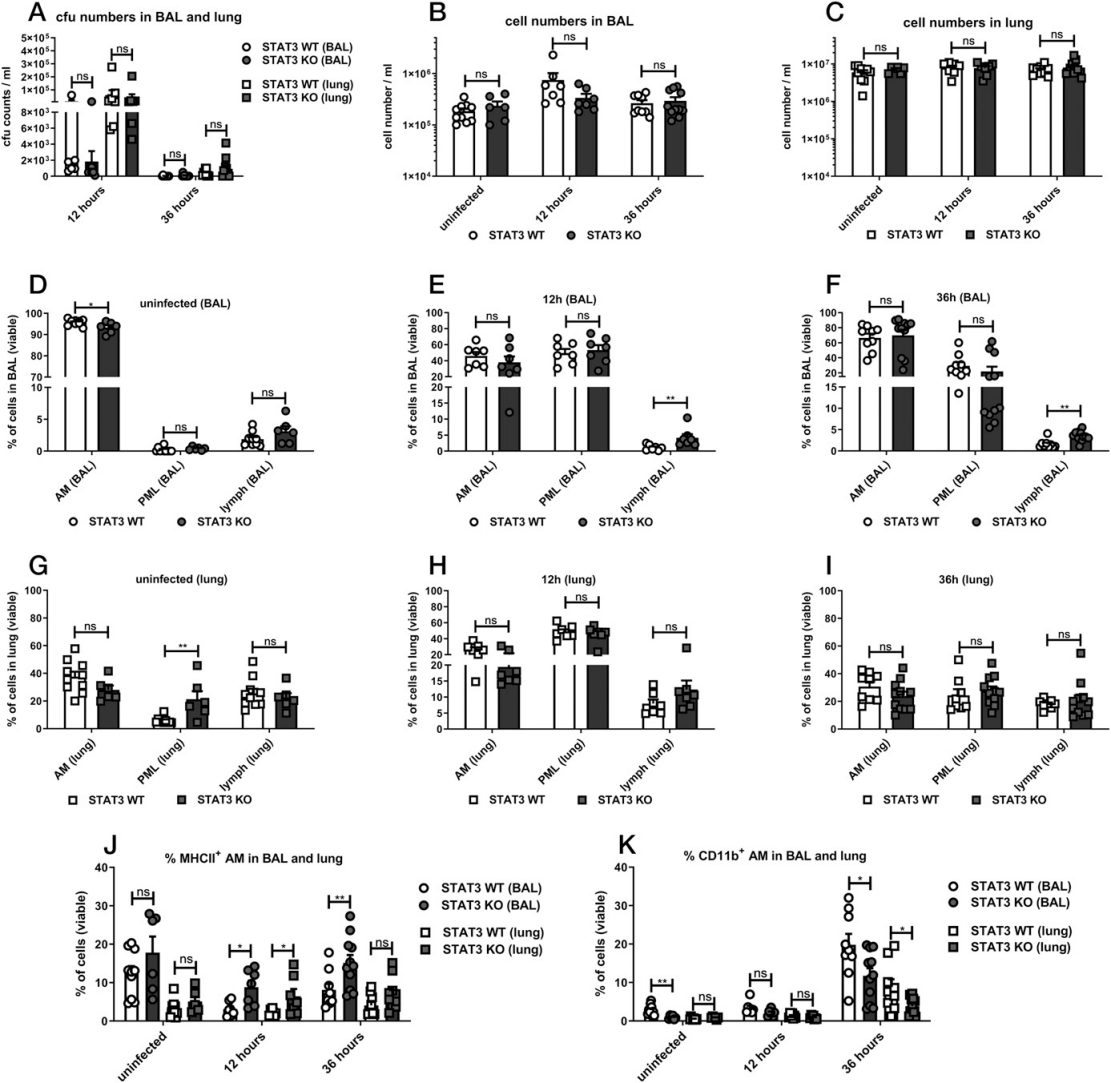
Major cell distributions in BAL and lung during intratracheal staphylococcal infection in mice with and without myeloid STAT3 deficiency. STAT3 KO and WT mice were infected intratracheally with a sublethal dose of S. *aureus* 8325-4 (∼5 × 10^6^ CFU) or an equivalent dose of PBS (uninfected). Mice were followed for 12 or 36 h. Then, BAL was performed, and lungs were harvested. (**A**) CFU counts were determined in BAL and lung cell suspensions via serial dilution plating prior to centrifugation and supernatant filtration. Supernatants were stored, and the remaining cell pellets were further processed by red cell lysis and washes. (**B** and **C**) Total cell numbers were counted with a cell counter. The cell suspensions were then further analyzed by multicolor flow cytometry. The gating strategy is displayed in Supplemental Fig. 2. (**D**–**I**) Display of AM, neutrophil granulocytes (PML), and lymphocytes (lymph) in (D, G) uninfected state, (E, H) at 12 h, and (F, I) at 36 h in BAL and lung, respectively. (**J** and **K**) MHCII and CD11b upregulation in AM was analyzed in more detail in the respective groups in the uninfected state, at 12 h, and at 36 h in BAL or lung cell suspension. Displayed are (J) MHCII^+^ AM and (K) CD11b^+^ AM in BAL and lung. Pooled data (mean with SEM) from *n* = 3 independent experiments are shown. A nonparametric Mann–Whitney test was used for statistical analysis. *p* > 0.05 not significant; *p ≤ 0.05, **p ≤ 0.01.

In order to study the effect of myeloid STAT3 deficiency in vivo, we used a nonlethal *S. aureus* pneumonia mouse model, where staphylococci were installed intratracheally. We observed that the bacteria had rapidly translocated to the lungs at 12 h after infection in both STAT3-deficient and WT mice. At 36 h, almost all animals had cleared *S. aureus* from their BAL and from the lungs, regardless of STAT3 (Fig. 4A). No statistical differences were observed regarding the number of bacteria (CFU) in BAL or in the lungs depending on STAT3. Although there were no differences in the overall immune cell counts between STAT3-deficient and WT mice (Fig. 4B, 4C), there appeared to be slightly fewer alveolar macrophages (AM) in the BAL of STAT3-deficient mice before infection (Fig. 4D). During infection, the percentage of AM and neutrophils (polymorphonuclear leukocytes [PML]) in the BAL did not differ at 12 or 36 h after infection (Fig. 4E, 4F). Yet, there was a tendency toward elevated lymphocyte numbers in the BAL from STAT3-deficient mice. PML numbers in the BAL were almost absent in uninfected animals, highest at 12 h, and then diminishing at 36 h, with no differences depending on STAT3. However, uninfected STAT3 KO mice showed increased neutrophil infiltration in the lungs (Fig. 4G). This difference disappeared after infection, and no differences were found in the cell distribution of AM, PML, or lymphocytes in the lungs of STAT3-deficient versus WT mice at 12 or 36 h (Fig. 4H, 4I). Enhanced expression of MHCII in AM was detected, though, in STAT3 KO as compared with WT mice, which was most evident in the BAL at both 12 and 36 h and in the lungs at 12 h (Fig. 4J). Compared with the STAT3-deficient mice, the percentage of CD11b**^+^** AM in the BAL of uninfected animals and in the BAL and lungs at 36 h after infection was higher in the WT mice (Fig. 4K).

### MMP9 expression is enhanced in inflammatory regions of the lungs in STAT3-deficient mice during staphylococcal pneumonia

**FIGURE 5.**
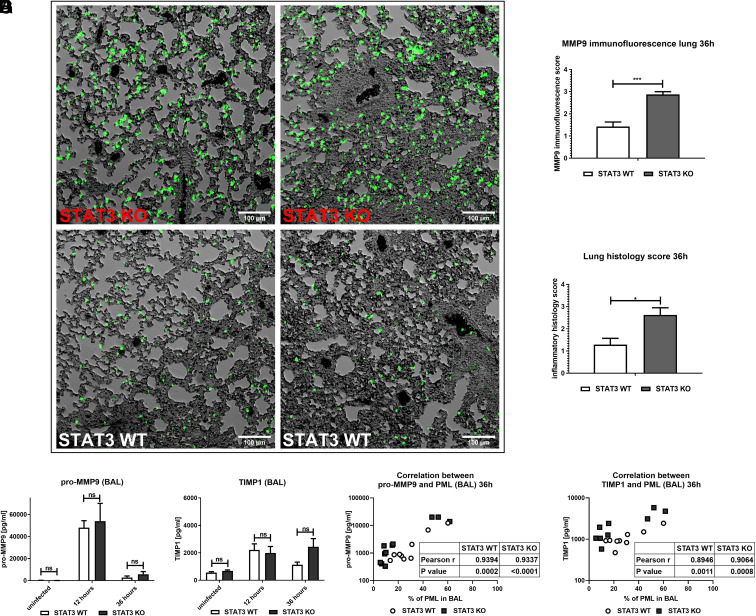
Augmented MMP9 expression in the lung during murine pulmonary *S. aureus* infection. STAT3 KO and WT mice were infected intratracheally with a sublethal dose of *S. aureus* 8325-4 (∼5 × 10^6^ CFU). Mice were followed for 12 or 36 h. Then, BAL was performed. In some experiments, lungs were removed and fixed in 10% formalin without prior performance of BAL. Samples were then embedded in paraffin. For microscopic analysis, sections were cut and put on slides. (**A** and **B**) Slide sections were subjected to immunofluorescence staining for MMP9 and analyzed with a DeltaVision microscope. Images were subjected to standardized analysis with macros in ImageJ. (A) Exemplary images of both STAT3 KO and WT lungs infected with *S. aureus* for 36 h (objective 20×, dimensions 2048 × 2048). (B) Nine images of each slide were taken, analyzed, and scored. (**C**) Routine histological H&E staining and histological scoring were performed by an independent pathologist in a blinded manner. The histological scores (B and C) were analyzed by nonparametric Mann–Whitney test. Pooled data (mean with SEM) from *n* = 2 independent experiments with three or more STAT3 WT/KO mice per group are shown. (**D**–**G**) BAL supernatants were analyzed by ELISA for (D) pro-MMP9 and (E) TIMP1 in uninfected mice and infected mice at 12 h and 36 h of infection. (**F** and **G**) Correlation analysis was performed between (F) pro-MMP9 and (G) TIMP1 levels and percentage of neutrophils in BAL at 36 h. Pooled data (mean with SEM) from *n* = 3 independent experiments are shown. A nonparametric Mann–Whitney test was used for statistical analysis of (B)–(E), and Pearson correlation analysis was performed to assess the correlation in (F) and (G). *p* > 0.05 not significant; *p ≤ 0.05, ***p ≤ 0.001.

In agreement with the results obtained from the in vitro findings in BMDM, there was an upregulation of MMP9 in the lung tissue of STAT3-deficient compared with WT mice as studied by immunofluorescence staining at 36 h (Fig. 5A, 5B). The enhanced signal was observed even in areas with similar cell infiltration. Lungs from STAT3-deficient mice displayed higher inflammatory scores than WT lungs at 36 h of infection (Fig. 5C). The overall cell distribution (AM, PML, lymphocytes) was, however, not significantly different at this time point (Fig. 4I). In BAL, similar detection of pro-MMP9 and TIMP1 was observed regardless of STAT3, and pro-MMP9 levels were considerably higher at 12 h than at 36 h (Fig. 5D, 5E), which was concomitant with higher PML numbers in the BAL at this time point (Fig. 4E, 4F). At 36 h, both enzymes correlated with the percentage of PML in BAL (Fig. 5F, 5G).

### The enhanced inflammatory phenotype observed in STAT3-deficient mice did not enhance the clearance of *S. aureus* as compared with WT

We did not find any differences in pathogen clearance depending on the presence of STAT3 in the pulmonary *S. aureus* mouse infection model (Fig. 4A). In vitro, we saw an effect of STAT3 neither on the number of internalized bacteria into BMDM nor on external *S. aureus* after 2 h of bacterial exposure (Supplemental Fig. 3C–3E). BMDM, regardless of STAT3, were inefficient in eliminating internalized *S. aureus*. At an MOI of 10, there was only an average 1-log decrease in remaining internalized bacteria after 24 h following elimination of all external bacteria at 2 h (Supplemental Fig. 3F). This means that both STAT3 and WT BMDM only killed ∼10% of the internalized bacteria after 24 h. STAT3-deficient and WT BMDM appeared morphologically affected and less activated when internalized *S. aureus* at 2 h was left viable for 24 h (Supplemental Fig. 3G, 3H). In contrast, microscopic imaging showed similar cell phenotypes and enhanced cell density at 24 h in LPS- and *S. aureus*–treated BMDM for both STAT3-deficient and WT mice, without major cell loss if bacteria were killed by addition of penicillin-streptomycin after 2 h (Supplemental Fig. 3I, 3J). No differences in cell death as measured by lactate dehydrogenase release were observed depending on STAT3 at either 24 or 48 h after infection within our infection model (Supplemental Fig. 3K, 3L).

### STAT3 deficiency leads to increased levels of miR-155 upon stimulation with *S. aureus*

**FIGURE 6.**
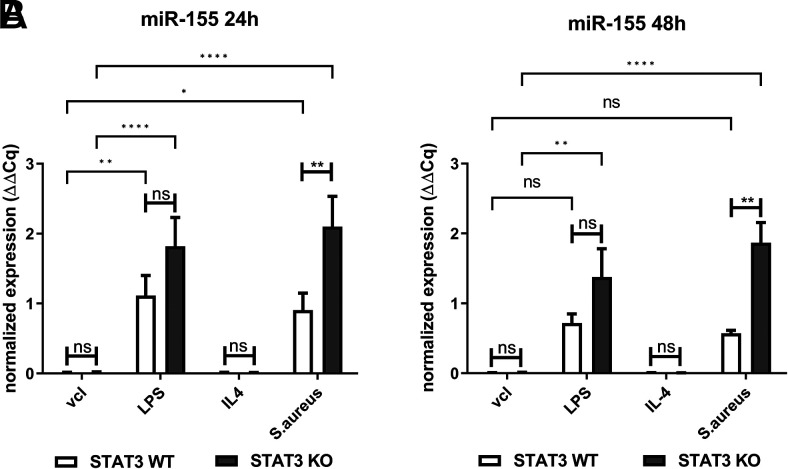
Lack of STAT3 strongly increases miR-155 expression upon stimulation with *S. aureus* in BMDM. BMDM from STAT3 WT and KO mice were stimulated with vcl, LPS (100 ng/ml), IL-4 (20 ng/ml), or *S. aureus* (MOI 10). At 2 h of stimulation, penicillin-streptomycin was added to kill off viable *S. aureus*. At 24 and 48 h, RNA was generated using the TRIzol method, and miR-155 expression was analyzed. (**A** and **B**) Normalized expression of miR-155 to sno202 at (A) 24 h and (B) 48 h. Pooled data (mean with SEM) from *n* ≥ 3 independent experiments are shown. Data were analyzed employing a mixed-effects model with Bonferroni analysis for multiple comparisons. *p* > 0.05 not significant; *p ≤ 0.05, **p ≤ 0.01, ****p ≤ 0.0001.

Recent studies, albeit mostly in cancer research, suggest multiple interactions between STAT3 with different microRNAs (27). Among these, miR-155 appears particularly interesting because it has been suggested to be involved in pulmonary inflammation and formation of the M1 macrophage phenotype, and it is also associated with functional IL-10 signaling (28). Thus, we assessed miR-155 expression at different time points in our in vitro model. miR-155 expression was increased in BMDM challenged with LPS or *S. aureus*, which was most pronounced at 24 h after infection (Fig. 6A). Lack of STAT3 further enhanced miR-155 expression, in particular in *S. aureus*–treated cells. Upregulation of miR-155 was also seen at 48 h in STAT3-deficient BMDM (Fig. 6B).

## Discussion

*S. aureus* is an intriguing pathogen because it may colonize the human host for long time periods but may also become pathogenic and cause life-threatening disease. Its mode of action depends on both bacterial risk factors and immune host functions (29, 30). Macrophage activation has recently been suggested to play a crucial role, in particular during the initial phase of staphylococcal infection, because it orchestrates the subsequent immune response by activating and connecting innate and adaptive immunity (31). In our in vitro infection model, we observed induction of a unique macrophage phenotype after *S. aureus* challenge, with several similarities with the M1 phenotype induced by LPS, such as production of the proinflammatory cytokines TNF-α, IL-12, and IL-6, known to stimulate epithelia, tissue, and other immune cells (32, 33). An M1-like phenotype is essential during the initial host defense (34), and macrophage depletion leads to deficient bacterial clearance in murine pulmonary *S. aureus* infection (35). However, an enhanced M1-like phenotype also results in tissue damage during bacterial infections (36), and a switch to an M2-like phenotype is needed to provide postinfectious healing. Notably, the promotion of the M2-like phenotype can ultimately also be (ab)used by the bacteria to promote their own survival and colonization (37). Thus, it is crucial to understand which factors may tip the scale between too much and too little inflammation.

Activation of BMDM may involve different pathogen recognition receptors (PRRs). Among the various PRRs involved in staphylococcal sensing, TLR2 may recognize a variety of staphylococcal pathogen-associated molecular patterns and is considered a key PRR in staphylococcal defense (38, 39). Interestingly, we found that *S. aureus* infection of WT BMDM strongly downregulated CD86 while concomitantly inducing a mainly proinflammatory cytokine response. CD86 and MHCII downregulation have previously also been described in human monocytes treated with heat-killed *S. aureus* and were assumed to depend on both TLR2 activation and IL-10 induction (40). Notably, we found that IL-10 was equally induced in LPS- and *S. aureus*–treated WT cells, but LPS treatment did not downregulate CD86. This suggests that the downregulation of CD86 observed in response to *S. aureus* in WT cells is specific for activation by *S. aureus* as opposed to the LPS activation via TLR4. In line with this, TLR2 activation by *S. aureus* has been observed to contribute to both pro- and anti-inflammatory responses (38, 41) and *S. aureus*–induced IL-10 induction in BMDM was dependent on TLR2 stimulation (42). In our model, the lack of STAT3 abolished CD86 downregulation by *S. aureus* and increased CD86 surface expression of LPS-treated BMDM despite elevated IL-10 levels, which may be explained by nonfunctional STAT3-associated IL-10 downstream signaling. Notably, infection-associated MHCII downregulation has also been partly attributed to IL-6 signaling in other models (43). Thus, enhanced MHCII expression in STAT3-deficient BMDM upon LPS and *S. aureus* treatment may be caused by both defective STAT3-dependent IL-6 and IL-10 signaling. Downregulation of costimulatory molecules by *S. aureus* has been suggested to negatively affect the T-cell response and thus promote recurrent infections (40). In contrast, expression of CD80 and CD86 has been associated with excessive T-cell activation and proinflammatory cytokine induction, reducing survival in murine pulmonary *S. aureus* infections (32). In our nonlethal staphylococcal pneumonia model, the lack of myeloid STAT3 did not affect pathogen clearance and overall cell counts. Yet, inflammatory scores in the lung tissue were elevated, MHCII**^+^** cells were increased, and a significant elevation of lymphocytes was observed. This is in line with an enhanced lymphocyte activation induced by the macrophage phenotype in myeloid STAT3-deficient mice as observed in other models (44).

The hyperinflammatory phenotype observed in our infection model during STAT3 deficiency did not affect pathogen uptake, intracellular survival, or overall pathogen clearance in vitro or in vivo. Thus, immune evasion strategies used by *S. aureus* were not counteracted by the enhanced M1 phenotype, and survival within the macrophages was still enabled (45). Notably, an average of 90% of the bacteria, which had entered the macrophages at 2 h, were still viable after 24 h independent of the STAT3 genotype. These infected macrophages may be the source for subsequent bacterial dissemination, as observed by others (35, 46–48). Therefore, staphylococcal clearance is not automatically associated with an M1-type like cytokine response, but other STAT3-independent changes are necessary to boost pathogen killing. This observation may be one additional explanation for why patients with STAT3-deficient HIES experience recurrent staphylococcal infections, even though their neutrophils function normally (49). Because vehicle or IL-4 treatment did not lead to relevant phenotypic differences, we confirm a substrate-specific role of STAT3 during macrophage activation without affecting general macrophage differentiation. Moreover, the lack of a particular effect on other “classical” M1 markers, including iNOS and CD80, as well as on IL-1β production, argues that STAT3 is involved in only some but not all inflammatory processes attributed to the M1 phenotype.

The proinflammatory phenotype seen in our infection model is in line with the proposed predominantly anti-inflammatory role of STAT3 in myeloid cells (50). Overwhelming inflammation, such as induction of TNF-α responses, has been reported to exert a negative effect on the lung tissue during staphylococcal pneumonia (51). Also, patients with STAT3-deficient HIES show defective lung tissue healing, which is a major determinant of morbidity and mortality by favoring secondary opportunistic infections (15). Elevated plasma levels of MMP9 in STAT3-deficient patients (25) and its role in degradation of extracellular matrix during tissue remodeling led us to hypothesize that MMP9 may be differentially expressed in myeloid STAT3-deficient as compared with WT mice during pulmonary staphylococcal infection. Indeed, *Staphylococcus*-induced MMP9 expression and release were markedly elevated during myeloid STAT3 deficiency both in vitro and in vivo. However, LPS only induced a minor pro-MMP9 release in BMDM in our model, likely because it requires IFN-γ for efficient trafficking in this context (22). *S. aureus* did not induce IFN-γ in our model, suggesting a lack of relevance for the observed differences between STAT3 WT and KO BMDM. Notably, an *S. aureus*–derived protease and NO have been shown to activate pro-MMP9 (52, 53), and inhibition of MMP9 release has been associated with protection against tissue damage in a model of hyperoxic lung injury (54). These observations underline the supposed biological relevance of the induced MMP9 levels observed in our model. Both under- and overexpression of STAT3 signaling may lead to unfavorable MMP9 release due to the special role of STAT3 in orchestrating both pro- and anti-inflammatory responses. In cancer studies, overexpressed STAT3 has been associated with IL-6–induced MMP9 release, which favors invasiveness/metastasis (55). In murine RAW264.7 macrophages, IL-6 has been found to induce MMP9 by both STAT3-dependent and STAT3-independent mechanisms (via MAPK^erk1/2^ activation) (56). In addition, IL-10 expression was determined to be a major negative regulator of MMP9 expression as well as an inducer of TIMP1, which may counteract MMP9 (26). In our in vitro model, both LPS and *S. aureus* were able to induce IL-6, TNF-α, IL-12p70, and IL-10, but only *S. aureus* led to the marked MMP9 upregulation, which was also noticed at lower levels in the WT cells. This argues that the observed induction of MMP9 in BMDM is not dependent primarily on the induced cytokines but requires *Staphylococcus*-specific cell activation. This is in line with a reduced MMP9 induction in STAT3-deficient fibroblasts upon TNF-α treatment alone (57).

In our in vivo model, PML, which are a key source of prestored MMP9 (58), appeared to be the major determinant of MMP9 levels in the BAL. This was particularly evident in the fact that the highest MMP9 levels were observed at 12 h when PML were most abundant. In contrast, it has been suggested that MMP9 release by macrophages requires both transcription and active secretion (22), which were both strongly enhanced in STAT3-deficient BMDM stimulated with *S. aureus*. Importantly, we observed a strong MMP9 response in the lung tissue of myeloid STAT3-deficient mice at 36 h, when bacteria had almost been cleared and no difference in the PML distribution was evident. TIMP1 expression was not differentially affected by the lack of STAT3 in BMDM or in the BAL. The increased induction of MMP9 ultimately promoted a strong and rising elevation of the pro-MMP9/TIMP1 ratio in vitro. These observations appear to be especially relevant for the phenotype because elevated MMP9 and MMP9/TIMP1 sputum levels have been correlated with pulmonary damage in patients with ciliary dyskinesia (59). Furthermore, an increased MMP9 and MMP9/TIMP1 ratio in the blood were associated with community-acquired pneumonia (60). Thus, our findings not only may play a role in the observed phenotype in STAT3-deficient patients with HIES but also could be further explored as a rationale in diagnostic testing for staphylococcal pneumonia.

STAT3 is involved in a major way in the anti-inflammatory response of IL-10, which protects against hyperinflammation (61). IL-10–dependent suppression of TNF-α and IL-6 is defective in STAT3-deficient murine BMDM (44) and STAT3-deficient human monocytes (62, 63), which is in line with the cytokine signature observed in our model. Although the major downstream signaling pathway of IL-10 is via STAT3, other STAT3-independent pathways do exist (e.g., activation of STAT1, STAT5, and PI3K) (64). The observed differences in kinetics are most likely related to a variable degree of STAT3-independent pathways taking over. These may be particularly relevant for the more rapid decrease of the TNF-α time course, where both an additional IL-10–mediated STAT3-independent suppression (65) and post-transcriptional regulation have been reported (66). Notably, pure IL-10 deficiency results in a different phenotype with predominantly colitis and no predisposition to *S. aureus* infection or changes in lung structure (67). Therefore, the lack of IL-10 signaling alone is insufficient to explain all phenomena in human STAT3 deficiency, in particular because the cell type–specific response of the ubiquitously deficient STAT3 signaling may affect the outcome differentially. In our infection model of myeloid STAT3 deficiency, autocrine IL-10 is most likely the major component of the defective inflammation loop leading to the hyperinflammatory macrophage phenotype. Because IL-10 has been shown to inhibit miR-155 expression in a STAT3-dependent manner in murine macrophages (28), we analyzed the expression of the microRNA miR-155 and found enhanced miR-155 expression upon *S. aureus* stimulation in STAT3-deficient BMDM. miRNAs are small, noncoding RNAs that are involved in gene expression control via their post-transcriptional targeting of mRNA (68). miR-155 is a particularly well-known proinflammatory modifier of inflammation, and it may be induced by both TLR2 and TLR4 through either MyD88 or TRIF–dependent pathways as well as by autocrine TNF-α release (69). Because miR-155 upregulation was observed for both LPS and *S. aureus* stimulation, it may be involved in concomitant inflammatory signature (e.g., enhanced IL-6, TNF-α, IL-12p70) in STAT3 deficiency, but not in the divergent responses such as the observed MMP9 upregulation. Although we only assessed miR-155 in vitro, others have shown that overexpression in murine staphylococcal pneumonia was associated with a proinflammatory phenotype (7). Interestingly, knocking out miR-155 was found to partially inhibit murine postinfluenza methicillin-resistant *S. aureus* (MRSA) lung infection by enhancing IL-17 and IL-23 responses (70). In children with MRSA pneumonia, miR-155 has been associated with a Th9/IL-9 response, and targeting of miR-155 was suggested to be a potential new strategy to treat MRSA (71). Thus, in the present study, we identify *S. aureus*–induced overexpression of miR-155 as another feature of STAT3 deficiency that, most likely via enhanced TNF-α and ultimately deficient IL-10 signaling, may contribute to local hyperinflammation. Together with the observed enhanced MMP9 release as well as other epithelial factors involved in extracellular matrix remodeling (57), this may affect healing in staphylococcal pneumonia in STAT3 deficiency. Further research should focus on the relevance of these factors during staphylococcal infection in humans, taking into account the cell type–specific response by ubiquitously deficient STAT3 signaling.

**FIGURE 7.**
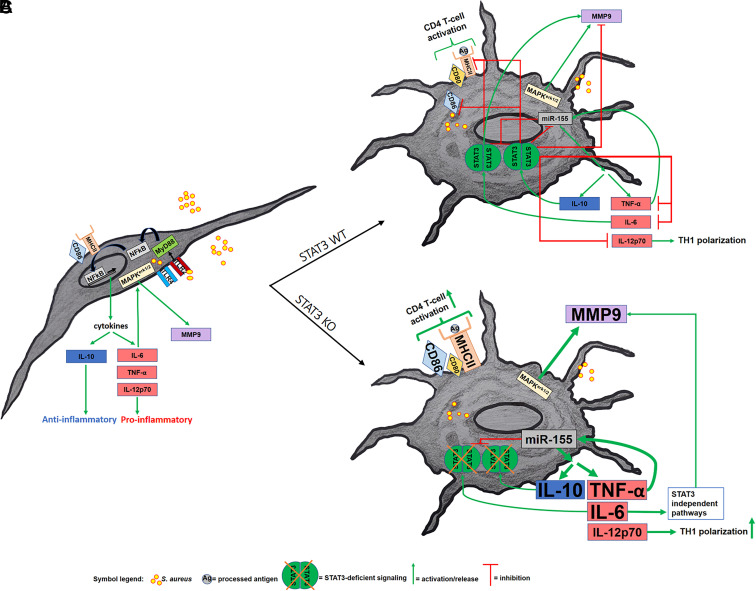
Simplified model of the inflammatory response of STAT3-deficient and WT BMDM to *S. aureus*. The figure summarizes the most relevant findings in our study and sets them into context of other pathways involved. (**A**) Nonactivated BMDM undergoing infection with *S. aureus* result in cytokine induction via TLR2, MyD88, and NF-κB. (**B**) *S. aureus*–infected and activated STAT3 WT BMDM exhibit an autocrine inhibitory IL-10/TNF-α circuit. (**C**) In STAT3-deficient BMDM, stimulation with *S. aureus* results in a hyperinflammatory phenotype with an increased induction of TNF-α, IL-12p70, MMP9, and miR-155, as well as elevated but ultimately nonfunctional IL-10 and IL-6, as compared with WT BMDM.

Overall, our data show that lack of STAT3 in murine BMDM shifts the macrophage phenotype to a hyperinflammatory phenotype during staphylococcal infection. Even though anti-inflammatory IL-10 is also elevated, it is inefficient because downstream signaling requires functional STAT3. Instead, the autocrine negative feedback loop is interrupted, and inflammation continues with STAT3-independent pathways likely taking over (Fig. 7). STAT3 is a relevant modifier of the inflammatory response of macrophages during staphylococcal infection, but not during general macrophage differentiation. Although macrophages do not greatly contribute to staphylococcal clearance themselves, they display a unique activation pattern when stimulated with *S. aureus*, which is markedly enhanced in STAT3 deficiency at several checkpoints. Apart from increased general inflammation, STAT3 deficiency does specifically boost MMP9 release and counteracts CD86 downregulation during *S. aureus* infection. The strong effect of STAT3 deficiency on the mainly proinflammatory response likely contributes to tissue damage and delayed healing in staphylococcal pneumonia in individuals with STAT3-deficient HIES.

## Supplementary Material

Supplemental 1 (PDF)
