# Supplementary material for: STAT3 Deficiency Alters the Macrophage Activation Pattern and Enhances Matrix Metalloproteinase 9 Expression during Staphylococcal Pneumonia
Source: J Immunol. 2023 Nov 20;212(1):69–80. doi: 10.4049/jimmunol.2300151 (PMC10733582; doi:10.4049/jimmunol.2300151)
Supplement: Supplemental 1 (PDF) [file JI_2300151_Supplemental_1.pdf]

Supplemental Figures

Supplemental Figure 1: Cell Purity, Exemplary Gating Strategy of BMDM in Flow Cytometry and Display of CD86 RNA Expression Induced by Stimulation of BMDM With Different Conditions

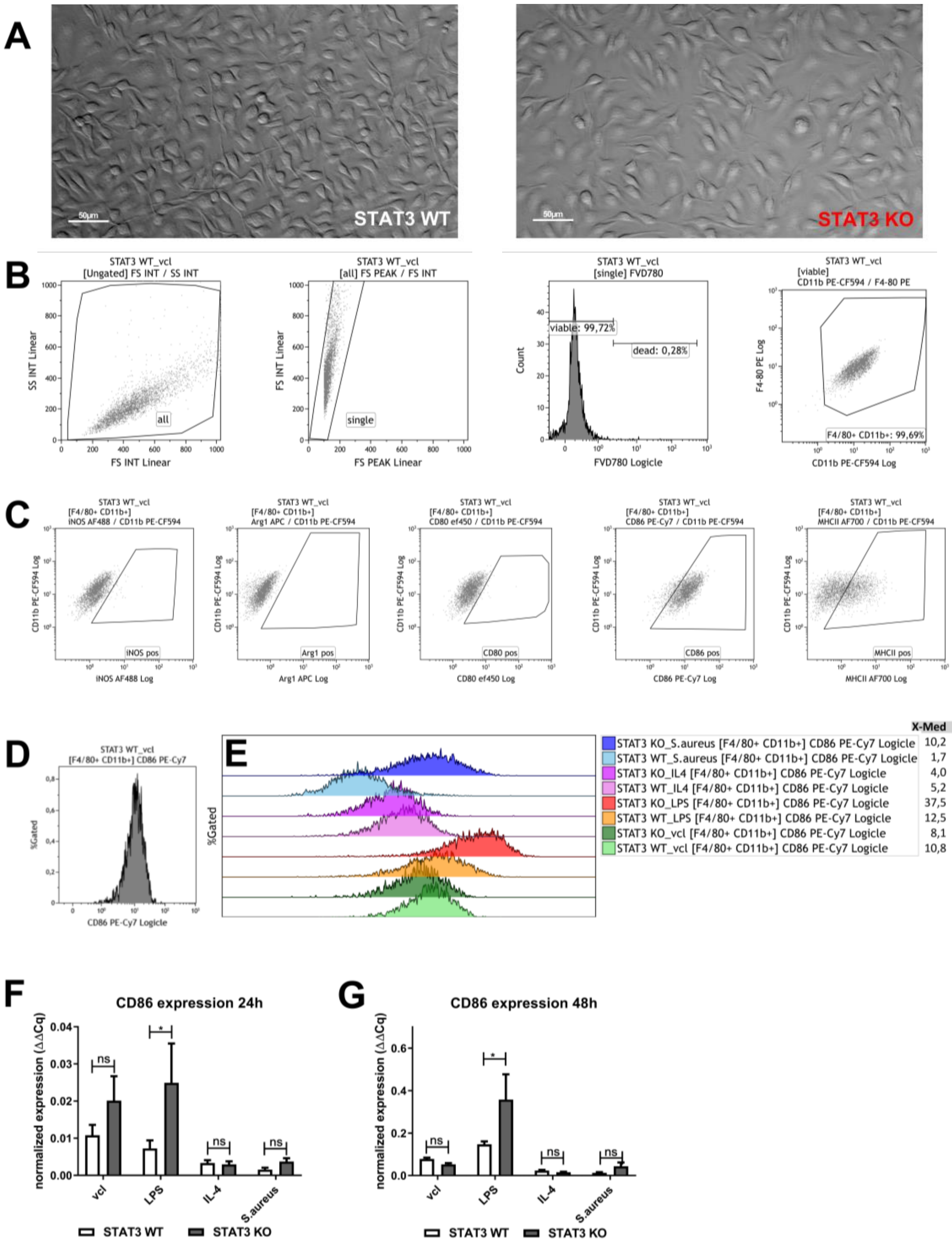

(A-G): BMDM from STAT3 WT and KO mice were stimulated with vehicle (vcl), LPS (100 ng/ml), IL-4 (20 ng/ml) or *S. aureus* (MOI 10) for a total of 24 or 48 hours following the addition of penicillin-streptomycin at 2 hours of stimulation. Supernatants were harvested and cells were processed for downstream analysis. **(A)** Display of BMDM morphology prior to stimulation in STAT3 WT and KO BMDM by light microscopy. **(B-E)** Flow cytometric analysis at 24 hours: Following live/dead staining (FVD780) and surface staining (CD80, CD86, MHCII), cells were washed, then fixed with 2% PFA and permeabilized, followed by staining for intracellular Arginase1 (Arg1) and iNOS. The figure exemplary visualizes the gating strategy on a STAT3 WT vehicle (vcl) sample. **(B)** Identification of F4/80^{+} CD11b^{+} BMDM by gating on viable, single-cell events. **(C)** Exemplary gating strategy in regards to the percentage of iNOS^{+}, Arginase 1 (Arg1^{+}), CD80^{+}, CD86^{+} and MHCII^{+} F4/80^{+} CD11b^{+} BMDM (=input gate) following 24-hour stimulation of STAT3 WT BMDM with vcl. **(D)** Exemplary display of a CD86 histogram of F4/80^{+} CD11b^{+} STAT3 WT BMDM treated with vcl. **(E)** Overlay of CD86 histograms for the different conditions (vcl, LPS, IL-4 and *S. aureus*) in one experiment and corresponding MFI (x-Med) as provided by Kaluza-software. **(F-G)** In selected experiments, cells were processed for RNA-extraction by TRIzol method. Following cDNA generation from RNA, qPCR of CD86 was run from **(F)** 24-hour and **(G)** 48-hour samples. Pooled data (Mean with SEM) from (**F**) n≥5 and (**G**) n≥3 different experiments are shown. Mixed-effect model with Bonferroni-Analysis for multiple comparison was used for statistical analysis ($p>0.05$=not significant (ns); $p\leq0.05$=*).

Supplemental Figures

Supplemental Figure 2: Intratracheal Staphylococcal Infection Model: Gating Strategy

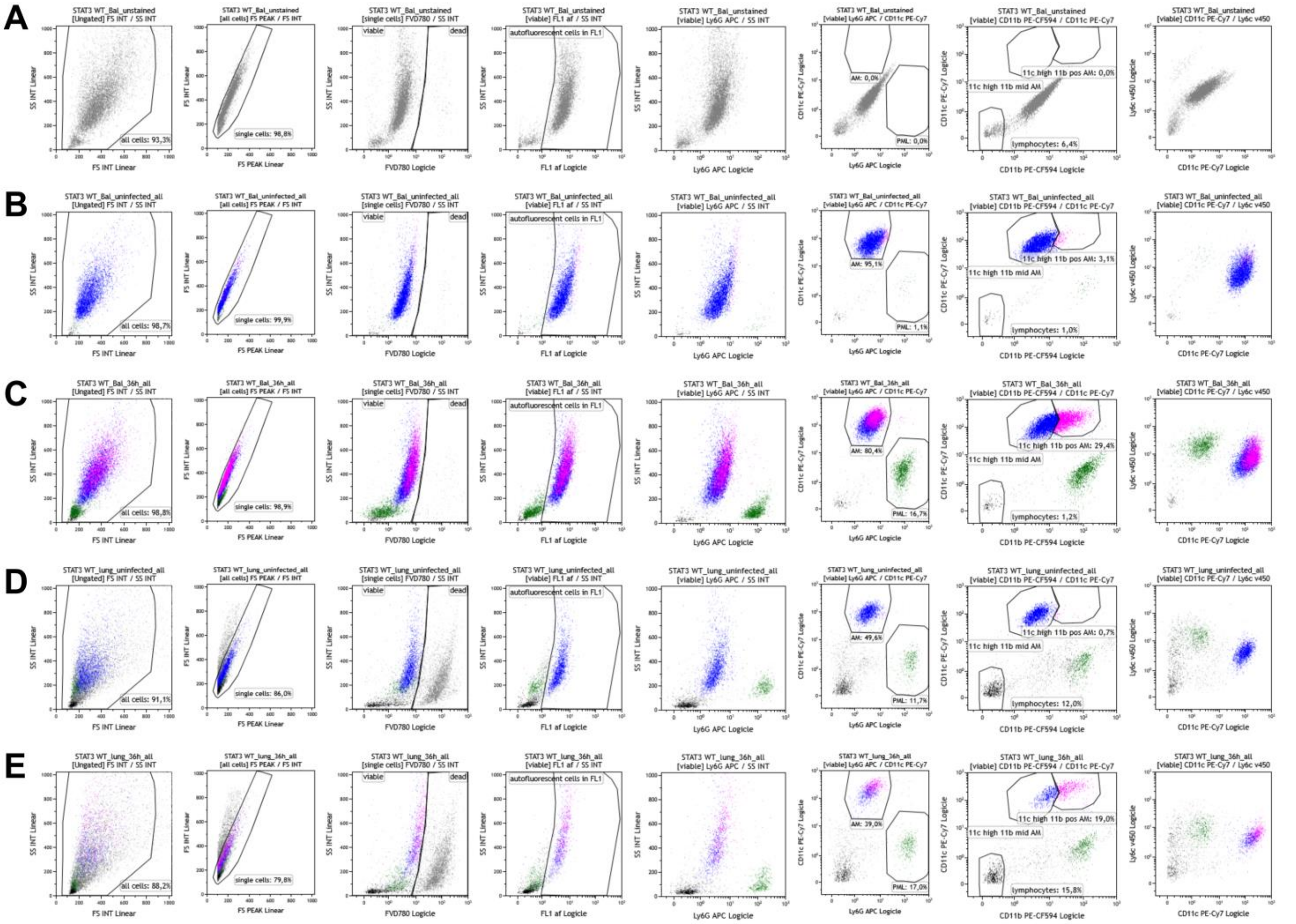

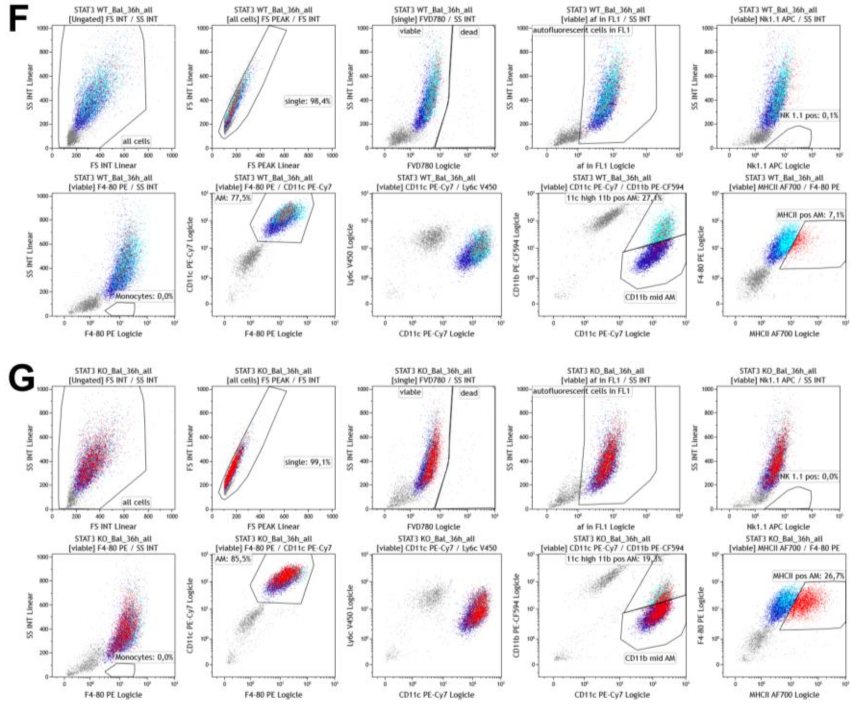

H

Identification of major cell populations:

Single, viable cell identification: FVD780$^-$ events within the single cell gate (FS int vs FS peak)

Alveolar macrophages (AM): high FSC/SSC, high autofluorescence (af) in FL1, CD11c high, CD11b mid (=position of CD11b positivity due to autofluorescence in this channel), F4/80$^+$

Neutrophils (PML): medium FSC/high SSC, low autofluorescence in FL1, Ly6G$^+$, Ly6C$^+$, CD11b$^+$

Lymphocytes (lymph): low FSC/SSC, no autofluorescence in FL1, CD11c$^-$, CD11b$^-$

Monocytes: middle-low FSC/SSC, CD11b$^+$, F4/80$^+$, Ly6C$^+$

MHCII positive AM: high FSC/SSC, high autofluorescence, CD11c high, F4/80$^+$ <u>and</u> MHCII$^+$

CD11b positive AM: high FSC/SSC, high autofluorescence, CD11c high, F4/80$^+$ <u>and</u> CD11b$^+$

(A-H) STAT3 KO and WT mice were anesthetized and subsequently infected intratracheally with a sublethal dose of S. *aureus* 8325-4 (ca. 5x10^6 cfu) or PBS (uninfected). At 12 or 36 hours, bronchoalveolar lavage (BAL) was performed. Afterwards, lungs were harvested. Flow cytometric cell analysis was performed on BAL and lung suspension samples after multicolor staining in different panels. **(A-E)** Exemplary display of gating strategy to identify major cell populations. Shown are **(A-C)** BAL and **(D-E)** lung samples derived from STAT3 WT mice: **(A)** BAL sample (unstained apart from FVD780), **(B)** BAL sample (uninfected), **(C)** BAL sample (36-hour infection), **(D)** lung sample (uninfected) and **(E)** lung sample (36-hour infection). **(F-G)** Exemplary display of Gating strategy to determine MHCII and CD11b state of AM as demonstrated in **(F)** a STAT3 WT and **(G)** a STAT3 KO BAL sample (both 36-hour infection). **(H)** Major cell populations.

Supplemental Figures

<u>Supplemental Figure 3</u>: Lack of STAT3 in BMDM Does Not Affect IL-1β Production, INF-γ Release, Phagocytosis, Staphylococcal Killing, or General Appearance of BMDM After Stimulation With *S. aureus* Within our Infection Model

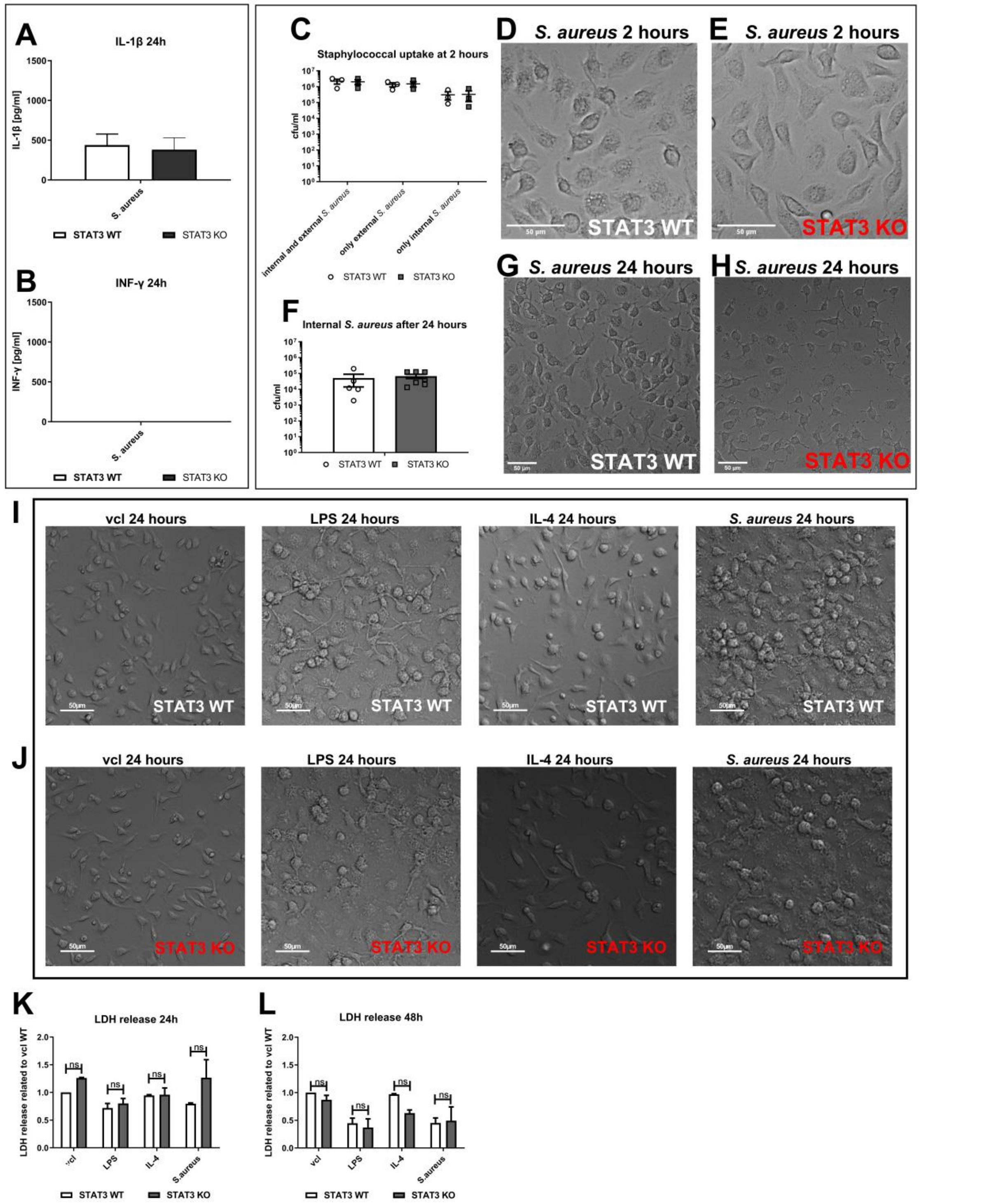

(A+B) IL-1β and INF-γ release was measured in supernatants 24 hours after stimulation of BMDM with *S. aureus* following the addition of penicillin-streptomycin at 2 hours of stimulation **(A) Lack of STAT3 in BMDM does not differentially affect IL-1β production of BMDM after stimulation with *S. aureus*** (Mean with SEM from n=5 experiments, detection limit 62,5pg/ml). **(B) No induction of INF-γ by S. *aureus* in our infection model at 24 hours** (n=4 experiments, detection limit 62,5pg/ml). **(C-H) Visualization of staphylococcal killing at 2 and 24 hours:** STAT3 KO and WT BMDM were incubated with *S. aureus* (MOI 10=5x10^6). **(C) Staphylococcal uptake**: gentamicin was added at 2 hours for 30 min to selected wells (=only internal *S. aureus*) followed by 2 washing steps in order to kill off and eliminate external bacteria. Then, 0,1% saponin was added to these wells and some wells without prior addition of gentamicin (=internal and external *S. aureus*) leading to lysis of BMDM. Subsequently, cfu counts were determined by serial plating of the supernatant of all wells, including other wells without saponin or gentamicin addition (=only external *S. aureus*) **(D+E)** Exemplary images (20x objective) of **(D)** STAT3 WT BMDM and **(E)** STAT3 KO BMDM stimulated with *S. aureus* at the 2-hour time point after wash and prior to lysis (image size 512x512)**. (F) Killing of internalized *S. aureus***: external *S. aureus* was killed with gentamicin at 2 hours of incubation. After a total incubation time of 24 hours 0,1% saponin was added. Subsequently, cfu counts were determined by serial plating. **(G+H)** Exemplary microscopic images of **(G)** STAT3 WT and **(H)** STAT3 KO BMDM from Experiment **F** at 24 hours prior to wash and cell lysis with 0.1% saponin (image size 1024x1024). **(I-J) Microscopic macrophage appearance of BMDM at 24 hours:** BMDM from STAT3 WT and STAT3 KO mice were seeded in 96 well microscopic plates and stimulated with vehicle (vcl), LPS (100 ng/ml), IL-4 (20 ng/ml) and *S. aureus* (MOI 10) for a total of 24 hours following the addition of penicillin-streptomycin at 2 hours of stimulation. At the 24-hour stimulation time point they were subjected to microscopy without prior fixation (20x objective, image size 896x896). Displayed are representative images of (**I**) STAT3 WT and (**J**) STAT3 KO samples treated with the respective conditions. **(K-L) Lack of STAT3 in BMDM does not enhance necrotic cell death of BMDM after stimulation with *S. aureus* in our infection model:** BMDM from STAT3 WT and STAT3 KO mice were stimulated with vehicle (vcl), LPS (100 ng/ml), IL-4 (20 ng/ml) and *S. aureus* (MOI 10) for a total of 24 or 48 hours following the addition of penicillin-streptomycin at 2 hours of stimulation. LDH release was measured at **(K)** 24 hours and **(L)** 48 hours and related to the values obtained in vehicle-stimulated (vcl) WT BMDM. Pooled data (Mean with SEM) from n≥2 independent experiments are shown. **Statistics**: Mixed-effect model with Bonferroni-Analysis for multiple comparison (p>0.05=not significant (ns)).

Supplemental Figures

Supplemental Table 1: Primer List

Target name	Description	*sense*	Sequence
**iNOS**	**Inducible nitric oxide synthase**	**forward**	ACCACTCGTACTTGGGATGC
		**reverse**	GTCTTGCAAGCTGATGGTCA
**β-actin1**	**Beta-actin1**	**forward**	GCAGGAGTACGATGAGTCCG
		**reverse**	AAACGCAGCTCAGTAACAGTCC
**Arg1**	**Arginase1**	**forward**	GTGAAGAACCCACGGTCTGT
		**reverse**	CCAGCTCTTCATTGGCTTTC
**CD86**	**CD86**	**forward**	TTGTGTGTGTTCTGGAAACGGAG
		**reverse**	AACTTAGAGGCTGTGTTGCTGGG
**B2M**	**Beta2-microglobulin**	**forward**	GCTACGTAACACAGTTCCACCC
		**reverse**	CATGATGCTTGATCACATGTCTC
**HMBS**	**Hydroxymethylbilane synthase**	**forward**	GAGTCTAGATGGCTCAGATAGCATG
		**reverse**	CTACAGACCAGTTAGCGCACATC
**MMP9**	**Matrix metalloproteinase 9**	**forward**	GCGGTCCTCACCATGAGTCC
		**reverse**	TGGAATCGACCCACGTCTGG
**Fizz1**	**Found in inflammatory zone 1**	**forward**	AGAGGTGGAGAACCCAGCTTTGAT
		**reverse**	TTTCAAGAAGCAGGGTAAATGGGCA
**Ym1**	**Chitinase-like 3**	**forward**	CAGGTCTGGCAATTCTTCTG
		**reverse**	GTCTTGCTCATGTGTGTAAGTG
**TIMP1**	**Tissue inhibitor of metalloproteinase 1**	**forward**	GCAAAGAGCTTTCTCAAAGACC
		**reverse**	AGGGATAGATAAACAGGGAAACACT

Primers were either adopted from previous publications or self-designed from DNA-sequences available at the Entrez Nucleotide database (https://www.ncbi.nlm.nih.gov). All primers were checked with BLAST for specificity and with the Mulitple primer analyser (ThermoFisher Scientific, www.thermofisher.com) for potential self- or oligodimers. In addition, primers were validated by performing a gradient-PCR. qPCR efficiency was between 92-108% for all primers listed. The following primer sequences were taken from literature: CD86, B2M (minor modifications), HMBS (minor modifications), Fizz1, YM1, TIMP1 (please contact the corresponding author for references).
